# A Joint Modelling Approach to Analyze Risky Decisions by Means of Diffusion Tensor Imaging and Behavioural Data

**DOI:** 10.3390/brainsci10030138

**Published:** 2020-03-01

**Authors:** Marco D’Alessandro, Giuseppe Gallitto, Antonino Greco, Luigi Lombardi

**Affiliations:** Department of Psychology and Cognitive Science, University of Trento, TN I-38068 Rovereto, Italy; giuseppe.gallitto01@gmail.com (G.G.); antonino.greco@unitn.it (A.G.); luigi.lombardi@unitn.it (L.L.)

**Keywords:** risk taking, diffusion tensor imaging, hierarchical Bayesian modelling

## Abstract

Understanding dependencies between brain functioning and cognition is a challenging task which might require more than applying standard statistical models to neural and behavioural measures to be accomplished. Recent developments in computational modelling have demonstrated the advantage to formally account for reciprocal relations between mathematical models of cognition and brain functional, or structural, characteristics to relate neural and cognitive parameters on a model-based perspective. This would allow to account for both neural and behavioural data simultaneously by providing a joint probabilistic model for the two sources of information. In the present work we proposed an architecture for jointly modelling the reciprocal relation between behavioural and neural information in the context of risky decision-making. More precisely, we offered a way to relate Diffusion Tensor Imaging data to cognitive parameters of a computational model accounting for behavioural outcomes in the popular Balloon Analogue Risk Task (BART). Results show that the proposed architecture has the potential to account for individual differences in task performances and brain structural features by letting individual-level parameters to be modelled by a joint distribution connecting both sources of information. Such a joint modelling framework can offer interesting insights in the development of computational models able to investigate correspondence between decision-making and brain structural connectivity.

## 1. Introduction

In cognitive neuroscience, relations between neural and behavioural characteristics of individuals are usually analyzed using a two-step approach which first summarizes performances on a given experimental task, and then applies standard statistical analysis on the neural and behavioural measures. However, several studies have highlighted the limitations of this approach in investigating and selecting theories to explain the relation between neural functioning and cognition [1,2,3].

Advances in the understanding of this relation are due to the development of different computational tools, allowing for a finer analysis of several sources of information. Some examples are: (1) cognitive modelling [4,5] which formally accounts for the generative cognitive processes which are assumed to produce the observed data; (2) Bayesian graphical models [6,7] which provide a powerful and flexible way to perform hierarchical Bayesian analysis, allowing to account for group and individual differences; (3) joint neurocognitive modelling [1,8,9,10,11] which provides a framework to simultaneously model and analyze neural and behavioural data by allowing the latter to be informative for the former, and vice versa.

The latter modelling framework has demonstrated to be an effective way to increase knowledge about the underlying neural substrates of cognitive functioning by bridging the gap between neuroscience and mathematical psychology. Here, the main advantage consists of using formal cognitive models as tools to isolate and quantify cognitive processes in order to effectively associate them with some brain measurements [8]).

In this work we aimed to put the emphasis on the mutual dependency between measures of structural integrity of brain regions of interest and cognitive functioning as assessed by the analysis of the outcomes of a given experimental task.

Several works ranging from perception [12], attention [13], memory [14], categorization [15], and decision in two alternatives forced choices [1,16] have demonstrated the need to formally account for reciprocal relations between mathematical behavioural models and brain functional or structural data.

In this contribution we proposed an architecture for jointly modelling such reciprocal relation in the context of risky decision-making. Although risk–decision tasks can be considered highly popular and effective experimental tools to investigate cognitive control and decision-making characteristics under risk conditions, a model-based approach to the joint analysis of brain and behavioural data in such contexts is still lacking.

Here, we proposed a novel way to relate structural information from Diffusion Tensor Imaging (DTI) to psychological parameters of a computational cognitive model accounting for the behavioural outcomes in the Balloon Analogue Risk Task (BART; [17]), from a confirmatory perspective.

The BART represents an ideal scenario to model decision-making since it has been correlated to “real-world“ risk taking [17,18]. The task has proven to reliably account for risk-taking propensity, response strategy and risk-related behaviour modulation in a broad range of normal and clinical populations [19,20,21,22]. In a typical BART setting, participants are required to decide whether to risk by inflating a balloon to earn a cumulative small monetary reward, being informed that the balloon might explode with a certain probability, thus causing the loss of accrued earnings. If participants decide to stop inflating they can cash out the current winnings. Optimizing total earnings in such a scenario is not trivial. In general, it requires a balanced risky-oriented strategy, learning from experience and modulating choices consistently [23].

In the present work we adopted a hierarchical Bayesian framework to relate neural and cognitive parameters inferred from performances of healthy participants on the BART. Analysis of posterior distributions was then used to assess relationships between the neural and cognitive variables. The model was applied to data from an already published dataset. Finally, the potentials in applying the method to the analysis of neural substrates underlying risk-taking behaviour and decision making were outlined and discussed.

## 2. Materials and Methods

### 2.1. The BART Data

The dataset used in this work was selected from the OpenfMRI database repository (http://www.openfmri.org; [24]) and refers to the experimental data reported in [25]. The dataset contains both behavioural performances and MRI scans from 24 healthy participants on a slightly modified version of the BART. Participants were adults recruited from UCLA’s campus with ages in the range 18–33, with no history of neurological illness and no use of psychoactive medication or illegal substances.

In the adopted version of the task, individuals saw a balloon on the monitor and were asked to select one of two possible options at each choice occasion for a given trial. The first option consisted in inflating the balloon, and is referred to as pump. The second option ended up the current trial by deciding to stop inflating the balloon, and is referred to as cash. Pumping the balloon increased the amount of possible monetary reward by 25 cents for each pump. If the participant decided to stop inflating the balloon, the accrued money was moved to a permanent store of winnings and a new balloon was presented. After a variable number of pumps the balloon exploded, in which case the participant lost all the money in the temporary pool. Participants did not receive any cue about the bursting probability. However, probabilities of explosions were not fixed and the actual number of pumps before an explosion followed a uniform distribution across trials, with an average of 6 pumps (SD=2 pumps). Each balloon was presented on each trial for a total of 36 trials.

### 2.2. The Cognitive Model

As previously outlined, performances of the BART are employed as a measure of risk-related behavioural tendencies and are usually analyzed by means of standard summary measures on test outcomes (e.g., total number of pumps, frequency of pumps across trials, number of cashes, number of explosions). However, such measures do not provide a suitable account for the data-generating process, that is, for the cognitive sub-processes involved in the task.

In this work we proposed a parsimonious computational account of the cognitive mechanisms underlying the observed response pattern of pumps and cashes [23,26]. In particular, we relied on a modified version of a robust model representation which has shown to be particularly stable to parameter recovery and estimation [23].

The model assumes a subjective probability estimate that a pump will make the balloon bursts in a given trial *k*. It also assumes that individuals determine the number of pumps for that trial prior to the first actions, and do not make adjustments during pumping. The number of pumps that individuals consider optimal on trial *k* is defined as $\omega_{k}$, and depends on the propensity of risk taking, γ, and on the current subjective bursting probability $p_{k}^{*}$, as follows:

(1) $$\begin{array}{ccc} \omega_{k} & = & -\frac{\gamma }{\log(1-p_{k}^{*})} \end{array}$$

where γ≥0. Equation (1) provides a parsimonious and effective representation of an individual decision strategy. Intuitively, $\omega_{k}$ places an upper bound on the pump attainable at a given trial, which is proportional to risk propensity, γ. The term $p_{k}^{*}$ in the denominator has the role of shrinking the number of pumps an individual considers as optimal. Moreover, the probability of pumping in trial *k*, at a given occasion *j*, is defined as $\theta_{kj}$ and depends on $\omega_{k}$ and on behavioural consistency, β, which can be though to account for response variability:

(2) $$\begin{array}{ccc} \theta_{kj} & = & [1+\exp{\left(\beta \left(j-\omega_{k}\right)\right]}^{-1} \end{array}$$

where β≥0. High values (resp. low values) of β mean less variable responding (resp. more variable responding). Equation (2) represents the fact that behaviour is generally determined by the divergence between the current choice occasion *j* (e.g., pump opportunity) and the optimal number of pumps, $\omega_{k}$. When the optimal number of pumps is exceeded ($j>\omega_{k}$ for the trial *k*), the probability of pumping, $\theta_{kj}$, approaches zero. However, parameter β reflects the degree to which a response is determined by such a divergence. When β=0, the individual decision to pump or cash is random. Differently, decisions become more consistently determined by the divergence criterion as β increases.

However, the original formulation of the model [23] assumed parameter $p_{k}^{*}$ to be fixed (which implies removing subscript *k*) and known from participants at the beginning of the task. This supports the assumption that the subjective probability of burst is constant across the task trials. However, fitting such model to our data could be problematic at least for two main reasons: (1) participants were not informed about the true bursting probability which in the task is uniformly distributed across trials; (2) in general, it is not possible to ensure that subjective bursting probabilities are consistent among participants and constant across trials, whatever the information they receive prior to the task.

In our model representation, subjective bursting probability and its dynamics were taken into account and inferred by relying on the history of participant’s choices. Let the variable $C_{k}$ indicate the cumulative success rate up to trial *k* according to:

$$C_{k}=\frac{\sum_{k=1}^{K-1}s_{k}}{\sum_{k=1}^{K-1}n_{k}}$$

where $s_{k}$ and $n_{k}$ are the number of successful (non-bursting) balloon pumps and total pumping attempts at trial *k*, respectively. Modelling $C_{k}$ as a Beta distributed random variable yields the statistical solution to the task of inferring the subjective bursting probability as follows:

$$\begin{array}{ccc} C_{k} & ∼ & \mathrm{Beta}(\mu_{\alpha_{k}}\sigma_{\alpha },\left(1-\mu_{\alpha_{k}}\right)\sigma_{\alpha }) \end{array}$$

(3) $$\begin{array}{ccc} \mu_{\alpha_{k}} & = & {\mathrm{logit}}^{-1}\left(\alpha_{0}+\alpha_{1}k\right) \end{array}$$

(4) $$\begin{array}{ccc} p_{k}^{*} & = & 1-\mu_{\alpha_{k}} \end{array}$$

where the cumulative success rate is regressed on trial numbers, and parameters $\alpha_{0}$ and $\alpha_{1}$, denoting the intercept and slope respectively, are the regression coefficients. For computational convenience we adopted the parameterization proposed by Ferrari and Cribari-Neto [27]. Such a parameterization has proven to be convenient in our computational setting since it allowed to model the observed cumulative success rate at trial *k* as sampled from a Beta distribution with expected value $\mu_{\alpha_{k}}$ and concentration $\sigma_{\alpha }$. Thus, the (conditional) expected value of the Beta distribution, given the specific trial, has been modelled as a function of cognitive parameters $\alpha_{0}$ and $\alpha_{1}$, and the specific trial *k*. The inverse logit function allowed to map such parameters to the natural domain of the expected value of the Beta distribution according to the specified parameterization. Here, $\alpha_{0}$ indicates the baseline subjective bursting probability and $\alpha_{1}$ represents the rate of change of bursting belief. In particular, if $\alpha_{1}<0$ (rep. $\alpha_{1}>0$), then this reflects an indicator that perceived bursting probability increases (resp. decreases) as the participant’s responses start accumulating balloon bursts as the trials unfold (resp., start decreasing balloon bursts).

At this point, the resulting cognitive model can be thought to account for response configurations of pumps and cashes by means of two hierarchically organized sub-models. In the first sub-model, a trial-specific bursting probability, $p_{k}^{*}$, is computed based on the baseline subjective bursting probability, $\alpha_{0}$, and the bursting belief dynamic yielded by $\alpha_{1}$. In the second sub-model, the decision process is instantiated by allowing the system to estimate an optimal number of pumps, $\omega_{k}$, conditioned on the computed trial-specific bursting probability, $p_{k}^{*}$, and a response is delivered based on $\theta_{kj}$.

Therefore, model representation allows to test the hypothesis that participants do not modify their initial bursting belief during the task. When $\alpha_{1}=0$, behaviour depends only on the baseline bursting probability and cognitive parameters γ, and β.

From a generative perspective, an observed pumping action $y_{kj}$ (1 if pump, 0 if cash) can be modelled according to a Bernoulli distribution:

$$\begin{array}{c} y_{kj}∼\mathrm{Bernoulli}\left(\theta_{kj}\right) \end{array}$$

and $\theta_{kj}$ depends on both cognitive parameters and the specific choice occasion within a specific trial. The likelihood function is then defined as follows:

(5) $$\begin{array}{c} p\left(Y|\Omega \right)=\prod_{k=1}^{K}\prod_{j=1}^{J(k)}\theta_{kj}^{y_{kj}}{\left(1-\theta_{kj}\right)}^{\left(1-y_{kj}\right)} \end{array}$$

where $\Omega =(\gamma ,\beta ,\alpha_{0},\alpha_{1})$ is the array of parameters of the behavioural model, and J(k) is the total number of observed actions for trial *k*.

### 2.3. The Neural Model

The cognitive model decomposition allowed to isolate individual cognitive characteristics and to rephrase them in terms of model parameters. A further step to model neural and behavioural data simultaneously consisted in bringing individual brain characteristics into the joint model. To this purpose, we focused on neural structural information at individual level. More precisely, we wanted the neural model to account for properties of structural connectivity in the brain. Consistently, we adopted Fractional Anisotropy (FA) as the founding measure to parameterize individual brain structural connectivity.

FA is the most commonly used index for estimation of anisotropy using DTI, and reflects fiber tracts characteristics such as the extent of alignment of cellular structures within the fibers and their structural integrity [28,29]. Therefore, such a measure also proved to be a promising index to study the relation between brain structural integrity and both response variability and risky behaviour in both clinical and normal population [19,30,31,32].

In this work, we were interested in relating cognitive functioning with connectivity measures of networks of regions of interest (ROIs). This choice was motivated by substantial evidences on the potential of functional-structural properties of distributed neural networks to account for complex decision processes [32,33,34]. Thus, the main purpose of our neurocognitive modelling approach consisted of linking connectivity-related information of network structures with the latent mechanisms captured by the computational cognitive model.

As a measure of connectivity we quantified the FA of white matter tracts relating specific regions of interest in a specific neural network. The confirmatory aspect of our approach was reflected by the choice of relying on a subset of the whole-brain structural connectivity matrix. To do this, a custom connectivity matrix was obtained by focusing on the following ROIs array and related white matter connectivity paths: left and right thalamus, striatum, dorsolateral prefrontal cortex, anterior cingulate cortex, inferior frontal gyrus, insular cortex. A network was then defined as the vector of elements of any (biologically) consistent subset of the ROIs array.

More formally, consider a square and symmetric connectivity matrix F, such that:

$$F=\left[\begin{array}{cccc} f_{11} & f_{12} & \ldots & f_{1J}\\ f_{21} & f_{22} & \ldots & \vdots \\ \vdots & \vdots & \ddots & \vdots \\ f_{I1} & f_{I2} & \ldots & f_{IJ} \end{array}\right]$$

where $f_{ij}=0$ for i=j, and I=J. The entries $f_{ij}$ specify the fractional anisotropy of the custom tract connecting ROIs *i* and *j*. We refer to Network FA [32] to represent structural connectivity in a given network. Thus, network FA consists of the collection of $f_{ij}^{\left(x\right)}$ such that i,j∈x, and x is the indicator variable reflecting the vector of ROI labels which constitute a defined network. Potentially, Network FA can be obtained for several combinations of ROI labels, and thus for several subsets of F.

**Figure 1:**
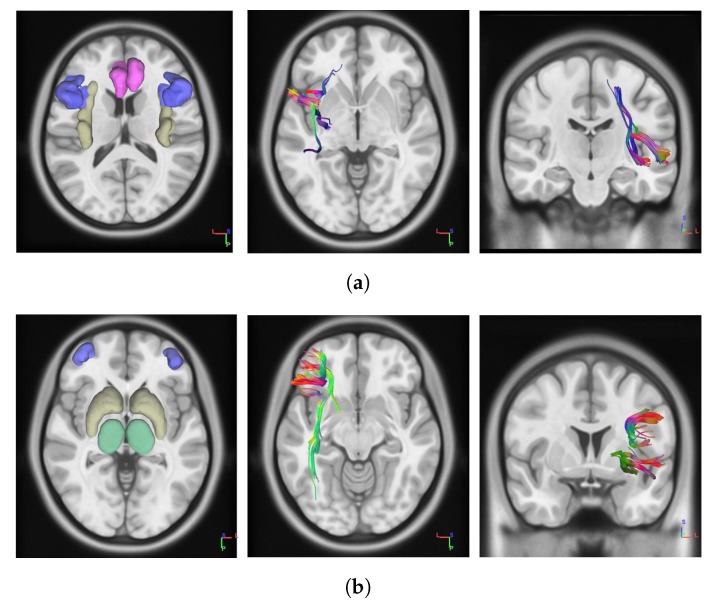
Pictures on the left show the regions of interest (ROIs) which constitute the networks. The network containing anterior cingulate (red), insula (yellow) and inferior frontal gyrus (blue) consists of the anterior cingulate cortex (ACC)–Insula–inferior frontal gyrus (IFG) Network (**a**). The network containing thalamus (green), striatum (yellow) and dorsolateral prefrontal cortex (dlPFC) (blue) consists of the dlPFC–Thalamus–Striatum Network (**b**). The central and rightmost pictures represent tracts of white matters connections for the first and the second network, respectively. For simplicity, figures show networks tracts for the left brain hemisphere, but the same applies to the opposite hemisphere. Network Fractional Anisotropy (FA) is intended to account for bilateral network tracts fractional anisotropy.

However, in this application we focused on two brain networks. The first network involves white matters connections between anterior cingulate cortex (ACC), insula, and inferior frontal gyrus (IFG), regions which are thought to be involved in loss-aversion modulations [33]. In particular, ACC is critically involved in cognitive control and decision-making processes in signaling anticipated risk and potential loss [35], whilst insula and IFG are thought to be implied in risk aversion signaling and risk avoidance during risky decisions [36,37]. The network implies fiber connections between ACC and IFG, and those implied by insular–cingulate and insular–frontal projections, bilaterally (Figure 1a). The second network involves projections between dorsolateral prefrontal cortex (dlPFC) and both striatum and thalamic nuclei, involved in top-down modulation of goal-directed behaviour [38] and, in part, in response variability in risky task [19]. Such a network is part of the Cortico–Striatal–Thalamic path, and consists of fiber connections between striatum and thalamus, and those implied by the striatal–dlPFC and thalamic–dlPFC projections, bilaterally (Figure 1b).

We refer to $\delta_{x=1}$ and $\delta_{x=2}$ as the neural parameters accounting for network FA measure for ACC–Insula–IFG and dlPFC–Thalamus–Striatum networks, respectively. Here, we followed a strategy proposed in [1] to easily provide a probabilistic account of the neural measures. In particular, tracts fractional anisotropy was assumed to be drawn from a Gaussian Distribution, which provided a computationally convenient and tractable probability model of Network FA:

(6) $$\mathrm{logit}\left(f_{ij}^{\left(x\right)}\right)∼\mathrm{Normal}\left(\delta_{x},\sigma_{x}\right).$$

Here, $\delta_{x}$ is thought to be the latent neural parameter accounting for structural property of network *x*, and $f_{ij}^{\left(x\right)}$ is the fractional anisotropy for tract connecting ROIs *i* and *j* in network *x*. Parameter $\sigma_{x}$ was thought to represent the inter-tracts variability of FA in the network *x*, and it was not conceived as accounting for Network FA since we were not interested in relating the variance in the inter-tracts FA measurements to cognitive parameters in the joint framework. However, it is worth noticing that such assumption might be infeasible when high inter-tracts variation is empirically detected, as in the case of measurements on the clinical population. In this case, more consistent parametric models might be considered to better account for Network FA.

The likelihood function is then defined as follows:

(7) $$p\left(f^{\left(x\right)}|\delta_{x},\sigma_{x}\right)=\prod_{n=1}^{N^{\left(x\right)}}N\left(\mathrm{logit}\left(f_{n}^{\left(x\right)}\right)|\delta_{x},\sigma_{x}\right)$$

where N(·) denotes the normal density with mean $\delta_{x}$ and standard deviation $\sigma_{x}$, and $N^{\left(x\right)}$ the number of connected tracts within the network *x*. We referred to $f_{n}^{\left(x\right)}$ as a simplified notation which reflects the FA value of the n−th tract connection between ROIs *i* and *j* in the network *x*.

Thus, neural parameters were inferred based on processed neural data and served to feed the system to account for the neural counterpart of the joint neurocognitive model, as will become clearer later in the next sections.

### 2.4. DTI Data Processing

The FA value computation was based on the eigendecomposition of the diffusion tensor [39]. In order to extract tracts’ FA, DTI diffusion images with a total of 64 volumes (diffusion sampling directions) with a b-value of $1000s/{\mathrm{mm}}^{2}$, in-plane resolution of 1.97917 mm and slice thickness of 2 mm, were used for analysis. All images have been corrected for eddy currents with FSL’s eddy toolbox using one b0 image as structural reference to account for geometrical distortions. The diffusion data were normalized in the MNI (Montreal Neurological Institute) space using affine registration and the ICBM-152 template, and a deterministic fiber tracking algorithm [40] was used. The tractography and connectivity matrix were calculated using DSI Studio (http://dsi-studio.labsolver.org). A seeding region was placed at whole brain and the fiber tracking procedure was performed with the thresholds of minimum FA value at 0.15, and maximum angle at $27^{\circ }$ according to previously utilized protocols [41]. The step size was randomly selected from 0.5 voxel to 1.5 voxels and tracks with length shorter than 30 or longer than 300 mm were discarded. A custom template with 12 ROIs (consisting of the brain regions whose tracts constitute F), six left and six right, was created using AAL2 [42], Desikan-Killiany-Tourville [43] and HCP842 [44] atlases and used as the brain parcellation. The connectivity matrix was calculated by using the FA of the connecting tracks.

### 2.5. Joint Modelling

A fundamental characteristic of joint models relies on their particular flexibility in allowing several assumptions about probabilistic (or deterministic) relations between neural and behavioural variables to be taken into account through model’s architecture.

In neurocognitive modelling are proposed two relevant architectures to account to the modelling of relationships between different sources of data: the *Directed Approach* and the *Covariance Approach* [11,45].

In the directed approach, a statistical model of neural data is defined and it is assumed that behavioural model parameters are directly affected by neural model parameters, codifying a non-reciprocal relation between the two sources of information. By contrast, the covariance approach does not assume such restrictions on parameters dependencies, but relies on specifying a joint model in which cognitive and neural parameters share a multivariate structure with covariance.

In this work, we adopted the latter as an adapted version of the joint model proposed by [1]. The primary reason for relying on the covariance approach was that we wanted to be agnostic in specifying the causal role of each source of information, that is, the directional statistical influence between neural and cognitive measures. To say it differently, the proposed covariance model combined both behavioural and neural models’ parameters in a unified framework, which characterizes the way behavioural and neural parameters coexist to explain the underlying cognitive process [45].

In our context, the covariance model has been thought to account for individual differences in task performances and brain structural characteristics by letting individual-level parameters to be modelled by a multivariate distribution connecting the two sources of information. Such connection allowed the information yielded by the neural data, as represented by F, to affect the information we learned about key cognitive parameters (e.g., γ, β).

We proposed a Multivariate Student’s t-distribution [46,47] as the multivariate probability model in order to account for robust relations between neural and cognitive parameters. Such relations were learned through hierarchical modelling and accounted by the (hyper-)covariance matrix of the multivariate distribution. Both cognitive and neural parameters were treated as latent variables.

For the behavioural model, we assumed structured individual differences in parameters γ, β, $\alpha_{0}$, and $\alpha_{1}$. The assumed model was also the one showing the best general fitting performances when compared to other possible models (see Supplementary Materials for model selection details). We further put a constraint on the relation between neural and cognitive parameters accounted by the covariance matrix of the multivariate probability model. In particular, we assumed that individual-level baseline bursting probability, $\alpha_{0}$ and its updating, $\alpha_{1}$, condition the behavioural model outside the covariance structure, and that risk-taking, γ and response variability, β, were then recovered within the multivariate probability model. To say it differently, we let $\alpha_{0}$ and $\alpha_{1}$ play the role of providing conditions for (possible) unbiased estimates of individual-level parameters γ and β, given that subjective probabilities have been taken into account.

**Figure 2:**
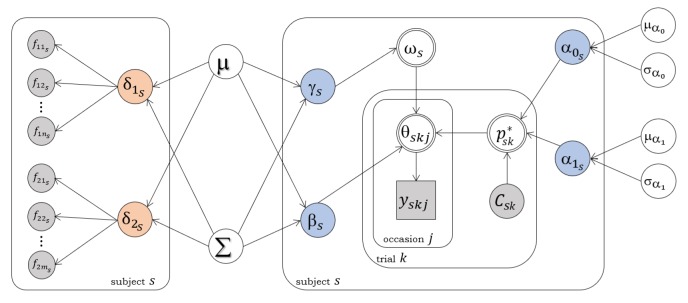
Covariance model’s architecture. Square and circular nodes indicate discrete and continous variables, respectively. Grey nodes indicate observed variables. Blue and red nodes represent behavioural and neural node parameters, respectively. Double-circled nodes represent deterministic nodes.

The Multivariate Student’s t-distribution was then specified by the hyperparameters vector:

$$\mu =\left(\mu_{\gamma },\mu_{\beta },\mu_{\delta_{1}},\mu_{\delta_{2}}\right)$$

containing the hyper-mean parameters for each of the individual-level parameters sharing a covariance matrix. The hyper-covariance matrix is thought to reflect research question and model assumptions. In our case we aimed to investigate the relation between pairs of network FA and cognitive sub-processes in a confirmatory perspective, and it was defined as follows:

$$\Sigma =\left[\begin{array}{cccc} \sigma_{\gamma }^{2} & 0 & \sigma_{\gamma }\sigma_{\delta_{1}}\rho_{1} & \sigma_{\gamma }\sigma_{\delta_{2}}\rho_{2}\\ 0 & \sigma_{\beta }^{2} & \sigma_{\beta }\sigma_{\delta_{1}}\rho_{3} & \sigma_{\beta }\sigma_{\delta_{2}}\rho_{4}\\ \sigma_{\gamma }\sigma_{\delta_{1}}\rho_{1} & \sigma_{\beta }\sigma_{\delta_{1}}\rho_{3} & \sigma_{\delta_{1}}^{2} & 0\\ \sigma_{\gamma }\sigma_{\delta_{2}}\rho_{2} & \sigma_{\beta }\sigma_{\delta_{2}}\rho_{4} & 0 & \sigma_{\delta_{2}}^{2} \end{array}\right]$$

where $\rho_{1}$ and $\rho_{2}$ account for the relation between risk-taking, γ, and both ACC–Insula–IFG and dlPFC–Thalamus–Striatum networks FA, $\delta_{1}$ and $\delta_{2}$, respectively. Correlation parameters $\rho_{3}$ and $\rho_{4}$ account for the relation between behavioural consistency, β, and both $\delta_{1}$ and $\delta_{2}$, respectively. Eventual relations between brain structural and cognitive characteristics were, thus, estimated on a model-based perspective. A graphical representation of the relation between the variables in the system is shown in Figure 2, which depicts the joint model’s architecture.

The graphical model represents all the (in)dependencies assumptions between the variables in the system. Given the model’s assumptions we can compute the joint posterior distribution of the model parameters conditional on observed data as follows:

(8) $$\begin{array}{cc} p(\delta_{1},\delta_{2},\Omega ,\mu ,\Sigma , & \sigma_{1},\sigma_{2},\mu_{\alpha_{0}},\sigma_{\alpha_{0}},\mu_{\alpha_{1}},\sigma_{\alpha_{1}}\left|Y,F\right)\propto \\ \; & \prod_{s}\left[p\left(Y_{s}|\gamma_{s},\beta_{s},\alpha_{0s},\alpha_{1s}\right)p\left(f_{s}^{\left(1\right)}|\delta_{1s},\sigma_{1}\right)p\left(f_{s}^{\left(2\right)}|\delta_{2s},\sigma_{2}\right)\right]\\ \; & \prod_{s}p\left(\delta_{1s},\delta_{2s},\gamma_{s},\beta_{s}|\mu ,\Sigma \right)p\left(\alpha_{0s}|\mu_{\alpha_{0}},\sigma_{\alpha_{0}}\right)p\left(\alpha_{1s}|\mu_{\alpha_{1}},\sigma_{\alpha_{1}}\right)\\ \; & p\left(\mu \right)p\left(\Sigma \right)p\left(\sigma_{1}\right)p\left(\sigma_{2}\right)p\left(\mu_{\alpha_{0}}\right)p\left(\sigma_{\alpha_{0}}\right)p\left(\mu_{\alpha_{1}}\right)p\left(\sigma_{\alpha_{1}}\right) \end{array}$$

where *s* represents individuals, and $f_{s}^{\left(1\right)}$ (resp. $f_{s}^{\left(2\right)}$) reflects the FA values for the brain tract connections in ACC–Insula–IFG network (resp. dlPFC–Thalamus–Striatum network) for individual *s*.

The first row on the right side of Equation (8) represents the likelihood of the joint structure which simultaneously includes behavioural and neural model likelihoods, the second row represents the related priors according to model factorization, that is, the multivariate probability model for the random vector of individuals neural and cognitive parameters and the probability models for the two regression coefficients. The third row specifies the hyper priors (see Appendix A for details). Such factorization allows computation of marginal posterior distributions via Markov Chain Monte Carlo algorithms (MCMC; [48]).

Parameter reflecting the degrees of freedom of the Multivariate Student’s t-distribution has been treated as a tuning parameter in order to ensure algorithm convergence and chains mixing (see Supplementary Materials for computational details).

## 3. Results

For the model fitting, one participant was excluded from the analysis due to corrupted and unreliable MRI scan. The joint model was then fitted to data from the remaining 23 participants. The data array consisted of the collection of pumps and cashes across trials and the custom structural connectivity matrix for each subject ($Y_{s},F_{s}$).

All calculations were performed with the aim of the efficient interaction between R [49] and JAGS [50] using the package “R2jags“ [51]. A probabilistic programming implementation (see Supplementary Materials for details) of the bayesian graphical model architectures was then provided and posterior distributions were computed using Gibbs Sampling algorithm [52]. We ran 12 chains of 15,000 iterations each, with a burn-in period of 5000 iterations and a thinning size of 1, parallelized on an Intel i7 6 cores CPU. Thus, we obtained 120,000 samples from the joint posterior. The total time required to perform the computation was about 35 minutes. Table 1 summarizes some of the posterior densities of interest.

**Figure 3:**
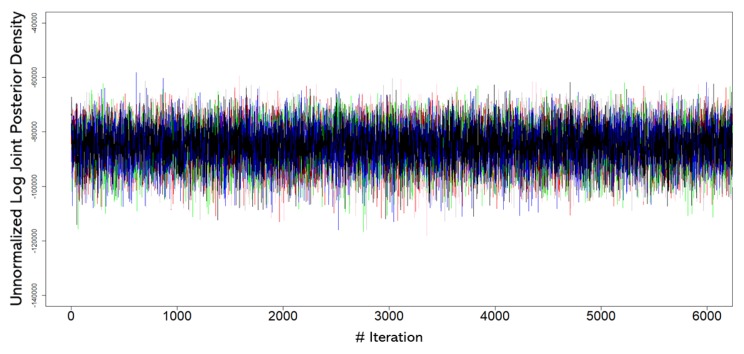
Trace plot of the (unnormalized) log posterior density computed for all the chains, for the first 6000 iterations. The burn-in period was removed to show the whole convergence dynamic. As can be noticed, the log posterior seems to show no trends.

Posterior marginals were sampled efficiently and the 12 chains showed an optimal convergence as measured by the $\hat{R}$ statistic [53], and the trace plot of the log joint posterior density (Figure 3). Values of $\hat{R}$ approaching 1 indicate better convergence.

**Figure 4:**
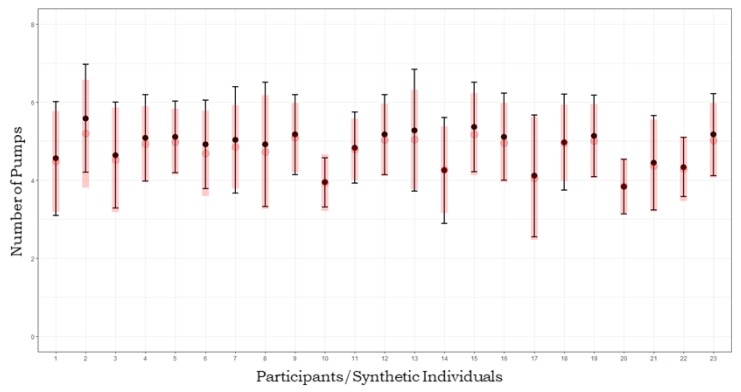
Posterior predictive check. Black dots and boundaries represent mean pumps and standard deviations for each individual from the empirical dataset. Red dots and lines represent mean pumps and standard deviations of predicted synthetic individual datasets.

Therefore, the joint model seemed to fit the data adequately by allowing a reliable recovery of cognitive parameters describing observed behaviour. Figure 4 shows results from posterior predictive check, which, in bayesian modelling, is the benchmark method to assess effective model fit [54]. We compared observed data to synthetic model-generated data produced by parameters drawn from the posterior distribution. Model fit adequacy was evaluated based on how much synthetic data resemble empirical data. We generated posterior predictives of 1000 datasets of pumps and cashes patterns on 36 trials, for 1000 cognitive parameter sets $\left(\gamma_{s},\beta_{s},\alpha_{0s},\alpha_{1s}\right)$ sampled from the joint posterior distribution corresponding to each individual *s*. Empirical distributions of number of pumps were then compared with recovered distributions.

Population (hyper-)means (see Supplementary Materials for Bayesian Sensitivity Analysis) allow to interpret individual differences in performance in terms of few parameters reflecting the assumptions about the process generating individual-level parameters [55].

At the population level, individuals seemed to modify their bursting belief only very slightly during the unfolding of the task (posterior mean $\mu_{\alpha_{1}}=-0.004$), and in general subjective bursting probabilities can be considered constant across the trials span. Therefore, individuals showed a relatively low level of risk-taking (posterior mean $\mu_{\gamma }=0.442$) and a relatively high level of behaviour consistency (posterior mean $\mu_{\beta }=1.471$) leading to low response variability.

**Figure 5:**
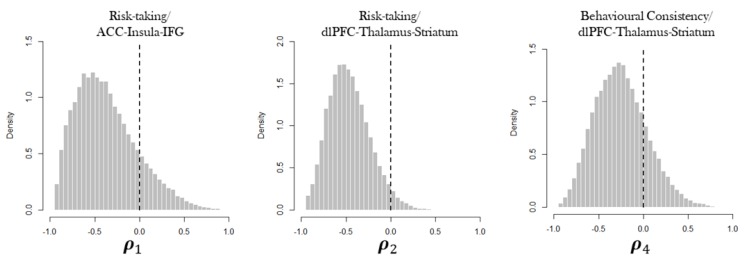
Marginal posterior distributions of the correlation parameters of interest in the covariance matrix.

In our confirmatory framework we aimed to verify whether such cognitive parameters configuration was related to Network FA. The multivariate distribution of the joint model allows to characterize such relation by treating groups of individual-level parameters as covariates. Thus, posterior densities of correlation parameters of the covariance matrix convey information on how individual differences in brain networks structural integrity and cognitive characteristics account for differences in performance. Figure 5 shows the estimated posterior distribution for the correlations between risk-taking, γ, and both ACC–Insula–IFG and dlPFC–Thalamus–Striatum networks FA, $\rho_{1}$ and $\rho_{2}$, respectively, and that between behavioural consistency, β, and dlPFC–Thalamus–Striatum network FA, $\rho_{4}$. The correlation between behavioural consistency and ACC–Insula–IFG is not shown since it has not substantial evidence.

In general, the relationships between parameters are weak, except for $\rho_{2}$, but the figure indicates a moderate inverse relation in all the three cases. Increase in risk-taking propensity was related to decreased white matter micro-structure integrity in two networks which codify for loss and risk aversion and for goal-directed behaviour. Such results might posit some constraints on the quantification of the actual role of risk propensity in enacting an optimal decision strategy. Participants were indeed required to adopt a balanced risky-oriented strategy in order to maximise earnings, and taking more risk at different stages of the task could be seen as an adaptive strategy which increases the chance to produce more positive outcome [32,56]. From this perspective, however, risk-taking propensity might not be the main component to fulfil the task of optimizing earnings. The finding of an inverse relation between behavioural consistency and dlPFC–Thalamus–Striatum network seems to clarify the role of white matter structural properties in predicting the adoption of a functional cognitive strategy when performing the BART. Individuals presenting an increased fractional anisotropy in such network showed an increased response variability (decreased behavioural consistency). This relation might reflect, in healthy individuals, the tendency to approach and explore the environment by choosing actions whose outcome is uncertain but potentially advantageous [19,57], as reflected by the functionality of the network.

## 4. Discussion

In the present work, we proposed an approach to the modelling of the neural structural substrates underlying risky behaviour within a joint modelling framework, inspired by previous works on joint analysis by means of hierarchical bayesian models [1,45]. A behavioural model allowing for estimation of meaningful cognitive parameters was developed and coupled with neural parameters in a multivariate probability model. This made the analysis of the relation between decision-making and brain structural connectivity interpretable on a model-based perspective.

The presented methodology application is thought to provide an example for cognitive scientists who are interested in investigating dependencies between behavioural and neural data via computational models. When applied to the experimental context of the BART, our approach shows several useful advantages.

First, the proposed computational framework is extremely flexible and has the potential to combine neural and cognitive models with several assumptions and complexities. This comes in handy when different BART configurations are considered (e.g., a priori knowledge of the bursting probability) and consistent behavioural model assumptions have to be made accordingly (e.g., removing the node related to the bursting belief dynamic, $\alpha_{1}$, from the graphical model).

Second, our method provides a way to infer the relationships between the biological properties of the brain and higher-level cognitive processes involved in risky decision-making by overcoming some limitations of the standard approach. For instance, inferences about the role of given cognitive mechanisms and their neural correlates in producing behavioural outcome, such as total monetary earning and relative frequency of pumps and cashes, implicitly assume a mapping between cognitive processes and summary measures of individuals task output. As a consequence, neurocognitive theories are built upon resulting relations between such statistics and brain measurements. Decomposing the data-generating process in several psychological sub-processes allows, instead, to relate brain measurements directly to cognitive variables of interest. In this respect, our computational model is valuable from a theoretical perspective since it can be used to test hypothesis about how neural variables predict cognitive functioning and behaviour in risk conditions, in a substantial formal way.

Therefore, the proposed architecture can be modularly extended to account for the presence of several explanatory variables. Thus, different covariates in the joint structure can be employed based on conditional dependence assumptions. As an example, one might think of explicitly modelling subjective bursting probabilities as predicted by discrete or continuous covariates such as sex, age, or self-report measures.

Moreover, several brain networks can be put in relation to cognitive variables of interest by extending the connectivity matrix F and the possible network subsets, and by extending the covariance matrix, Σ, to include more correlation parameters on a model-based perspective.

Despite these advantages, the proposed computational framework has some limitations. A first technical limitation might concern the fact that posterior probability computations become demanding and potentially unstable when the number of free parameters in the covariance matrix of the multivariate neurocognitive probability model increases. However, the Multivariate Student’s t-distribution adopted in the proposed application has shown to overcome some computational problems by ensuring posterior sampling chains mixing.

Second, our joint modelling framework assumes a previously defined parametric representation of the multivariate model accounting for cognitive and neural parameters. This might constitute a severe constraint since a probabilistic model describing neural data may not always be consistent, especially when complex brain structural measures, such as those provided by DTI, are considered.

It is worth noticing that other neural measures might be adopted to parameterize individual structural brain connectivity, and that embedding such measures in a joint structure could be far from trivial. In our model we adopted FA as an exemplary application, due to its popularity and (relatively) ease in being reliably computed. However, a more complex and exhaustive neural measure accounting for white matter anisotropy might be the Generalized Fractional Anisotropy (GFA, [58]). This is computed by using a more complete information on diffusion sampling directions and yields anisotropy maps with a higher angular resolution that might efficiently replace FA. Nevertheless, GFA estimates are particularly sensitive to noise and probably unreliable unless high angular sampling is available [59]. For this reason, a more accurate account of noise estimates has to be considered and formally instantiated in the joint graphical model, especially in relation to the covariance structure which directly relate neural and behavioural parameters.

In conclusion, we think that the proposed approach offers interesting insights in the development of computational models able to investigate correspondence between decision-making and brain structural connectivity. Further works are needed to investigate the potentials of the joint framework to account for BART performances and neural characteristics of individuals in clinical populations.

## Figures and Tables

**Table 1:** Marginal posterior distributions statistics: Posterior mean ($\mu_{post}$), 95% credible intervals $\left[q_{0.05},q_{0.975}\right]$, chains convergence ($\hat{R}$).

Parameter	$\mu_{\mathrm{post}}$	$q_{0.05}$	$q_{0.975}$	$\hat{R}$
$\mu_{\gamma }$	0.442	0.374	0.474	1.012
$\mu_{\beta }$	1.471	1.211	1.571	1.013
$\mu_{\alpha_{0}}$	2.653	2.460	2.722	1.001
$\mu_{\alpha_{1}}$	−0.004	−0.007	−0.001	1.001
$\rho_{1}$	−0.341	−0.85	0.365	1.019
$\rho_{2}$	−0.483	−0.86	0.072	1.010
$\rho_{3}$	0.021	−0.645	0.750	1.013
$\rho_{4}$	−0.250	−0.761	0.371	1.008

## Supplementary Materials

The following are available online at https://www.mdpi.com/2076-3425/10/3/138/s1.

## Abbreviations

The following abbreviations are used in this manuscript:

DTI	Diffusion Tensor Imaging
BART	Balloon Analogue Risk task
MNI	Montreal Neurological Institute

## Appendix A

The following probability distributions were used for the hyper priors:

$$\begin{array}{ccc} \mu_{\gamma },\mu_{\beta } & ∼ & \mathrm{Normal}{\left(0,10^{3}\right)}_{I(0,\infty )}\\ \mu_{\delta_{1}},\mu_{\delta_{2}} & ∼ & \mathrm{Normal}(0,10^{3})\\ \sigma_{\gamma },\sigma_{\beta } & ∼ & \mathrm{Gamma}(0.01,0.01)\\ \sigma_{\delta_{1}},\sigma_{\delta_{2}} & ∼ & \mathrm{Gamma}(0.01,0.01)\\ \sigma_{1},\sigma_{2} & ∼ & \mathrm{Gamma}(0.01,0.01)\\ \rho_{1},\rho_{2},\rho_{3},\rho_{4} & ∼ & \mathrm{Uniform}(-1,1)\\ \mu_{\alpha_{0}} & ∼ & \mathrm{Normal}{\left(0,10^{3}\right)}_{I(0,3)}\\ \sigma_{\alpha_{0}} & ∼ & \mathrm{Gamma}(0.01,0.01)\\ \mu_{\alpha_{1}} & ∼ & \mathrm{Normal}{\left(0,1\right)}_{I(-0.2,0.2)}\\ \sigma_{\alpha_{1}} & ∼ & \mathrm{Uniform}(0,1) \end{array}$$
