# Supplementary material for: A Joint Modelling Approach to Analyze Risky Decisions by Means of Diffusion Tensor Imaging and Behavioural Data
Source: Brain Sci. 2020 Mar 1;10(3):138. doi: 10.3390/brainsci10030138 (PMC7139494; doi:10.3390/brainsci10030138)
Supplement: Supplementary file 1 [file brainsci-10-00138-s001.pdf]

A Joint Modelling approach to analyse risky decisions by means of Diffusion Tensor Imaging and Behavioural Data:

Supplementary Material

Department of Psychology and Cognitive Science

University of Trento

Marco D'Alessandro

Giuseppe Gallitto

Antonino Greco

Luigi Lombardi

February 21, 2020

Send all correspondence to:

Marco D'Alessandro

Department of Psychology and Cognitive Science

Corso Bettini 31

I-38068 Rovereto (TN), Italy

Email: marco.dalessandro@unitn.it

1 BART behavioural Model Selection

In this section we compare 4 behavioural models accounting for different assumptions and cognitive parameters involved in the pattern of pumps and cashes produced by individuals.

As outlined in the main text, the BART version considered in our exemplar application did not provide participants any cue about the bursting probability. Probability of explosions was sampled for each trial, k, according to a uniform distribution.

The 4 models considered are the following: (1) a model assuming structured individual differences in three parameters, namely, γ, β and p, where p^* is a fixed bursting belief. Here, parameter p^* assumes a fixed and known bursting probability by removing the dependency on the specific trial k; (2) a model assuming structured individual differences in three parameters, namely, γ, β and α_0. The latter indicates the baseline bursting probability. Parameter α_0 is inferred based on the beta regression parameterization outlined in the main text; (3) a model assuming structured individual differences in four parameters, namely, γ, β and α_0, and a group-level parameter α_1 accounting for the dynamics of the subjective bursting belief; (4) the last model is the four parameter model used in the main text, which assumes structured individual differences for all the four parameters.

We assess models' performances by relying on both the *Deviance Information Criterion* (DIC) and the (mean) $\hat{R}$ statistic for each parameter across all individual estimates. Results are shown in the following table.

Model	DIC	Parameters	$\hat{R}$ (mean)
1	2071.3	γ	2.368
		β	1.001
		p^*	2.551
2	-1497.6	γ	1.001
		β	1.001
		α_0	1.001
3	-1531.6	γ	1.001
		β	1.001
		α_0	1.001
		α_1	1.001
4	-1595.6	γ	1.001
		β	1.002
		α_0	1.023
		α_1	1.005

As can be noticed, Model 4 has the lowest DIC, and $\hat{R}$ approaching 1, and can be selected as the best model.

2 Multivariate Model tuning in JAGS

In this section we discuss some computational details related to the tuning of the Multivariate Student's t-distribution. More precisely, we focus on the estimation of the correlation parameters in the covariance matrix of the multivariate model.

In our model representation, we assumed a sparse covariance matrix in which some correlation parameters were fixed to zero since our confirmatory approach focused on testing specific meaningful relations between cognitive and neural parameters. Thus, prior distributions should be considered for each correlation coefficient in the decomposed covariance matrix.

The JAGS probabilistic programming framework allows to embed prior distributions in the

hierarchical model by considering the Multivariate Student's t-distribution as modelled according to a precision matrix instead of a covariance matrix. Matrix inversion is then needed when correlation coefficients have to be obtained. However, matrix inversion problems may arise when the assumption of positive-definite matrix is violated, and it is often the case in which this happens.

Differently, prior distributions for the covariance matrix parameters can be specified when the Multivariate Normal distribution is considered. This allows to estimate correlation coefficients when the Multivariate Student's t-distribution is taken into account by overcoming the limitations related to matrix inversion.

Consider the vector-valued random variable $\boldsymbol{X^*} = [\gamma^*, \beta^*, \alpha_0^*, \alpha_1^*]$. We assume $\boldsymbol{X^*}$ to have a Standard Multivariate Normal distribution:

$$\boldsymbol{X^*} \sim N(\mathbf{0}, \boldsymbol{\Sigma})$$

with 4-dimensional zero mean vector and covariance matrix $\boldsymbol{\Sigma}$ as structured according to the main text. Consider now the vector-valued random variable $\boldsymbol{X} = [\gamma, \beta, \alpha_0, \alpha_1]$ containing the individual level cognitive and neural parameters. We model $\boldsymbol{X}$ as a Multivariate Student's t distributed random variable as follows:

$$\boldsymbol{X} = \boldsymbol{\mu} + \boldsymbol{X^*} \left(\sqrt{\frac{\zeta}{V}} \right)$$

where $\boldsymbol{\mu} = [\mu_\gamma, \mu_\beta, \mu_{\delta_1}, \mu_{\delta_2}]$ and V is a Chi-square distributed random variable with parameter ρ denoting degrees of freedom. Note that parameters $\boldsymbol{\mu}$ and $\boldsymbol{\Sigma}$ are estimated in a hierarchical bayesian framework in the main text.

Here, ζ was fixed to a default value such that $\zeta = 5$. In general, values of ζ in the range [5-30] do not compromise posterior sampled chains mixing in our application. However, when ρ is treated as a parameter to estimate and left free to vary within a broader range, chains mixing is not ensured and parameter estimates are unreliable.

3 Simulation Study

In this section we provide a simulation study aimed to explore model performance in meaningful scenarios. In particular, a Monte Carlo 3-factorial design is employed to recover Effective Sample Size of posterior MCMC samples and Computation Time across levels of three factors, namely, Number of subjects, Number of ROI-to-ROI connections (which we refer to as ROIs), and the tuning scenarios. In particular, in each cell of the factorial design, parameters are sampled from the prior, synthetic neural and behavioural data are simulated based on the sampled parameters, and the simulated data pattern is used to fit the joint model. For each cell, the process of data simulation and model fitting is repeated for 10 times. Number of subjects are allowed to vary across three levels, that is, $[10, 30, 50]$, whilst the Number of ROIs across two levels, that is, $[5, 10]$. Simulating neural data consists in directly sampling an FA measure related to a specific ROI-to-ROI connection. The tuning factor consists in two levels in which the degrees of freedom of Multivariate t-distribution is fixed to a default value such that $\zeta = 5$, or is treated as a free parameter with an exponential prior such that $\zeta \sim \text{Exp}(1/30)$. Such a factorial structure entails a configuration of 12 cells, and a total of 120 data simulations and model fitting. To make the computations feasible we adopt a simplified version of the joint model in which the behavioural model consists only in parameters γ and β, risk taking and response variability, respectively. The remaining nodes of the graphical model remain unchanged.

As in the main paper, the calculations are performed in R, and MCMC samples are obtain via Gibbs Sampling. For computational convenience 6 chains of 5000 iterations each are used, with a burn-in period of 500 iterations and a thinning size of 1. Computations are parallelized on an Intel i7 6 cores CPU. The total factorial design simulation time was 14 hours.

The mean computation time to estimate parameters of the joint model within each cell of the factorial design is reported in Figure 1.

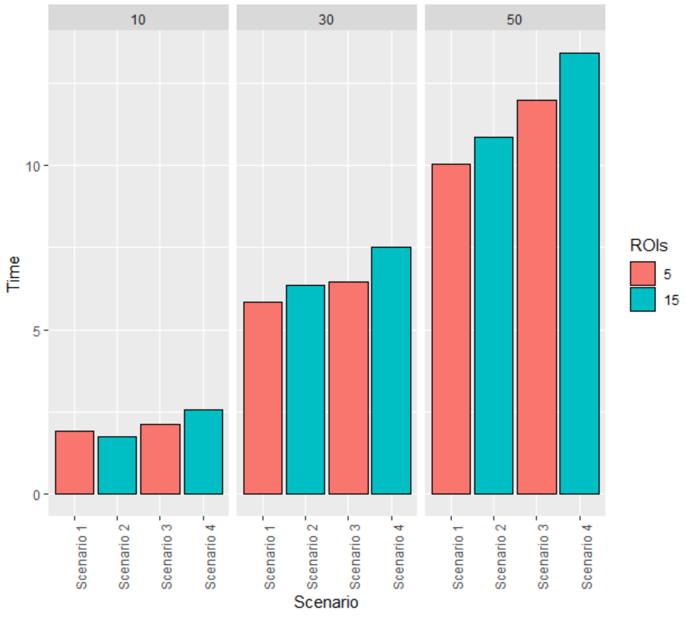

Figure 1: Mean computation time (in minutes) for each cell of the factorial design. Here, scenario 1 and 2 refer to the case in which ζ is fixed, whilst scenario 3 and 4 refer to the case in which ζ is treated as a free parameter.

As can be noticed, computation time increases slightly linearly, based on the number of individuals. Increasing the number of ROIs seems to contribute to extend the computation time especially for higher sample sizes. Scenarios in which ζ is treated as a free parameter (scenario 3 and 4) are the most computationally expensive. It is worth noticing that, in general, computations are rather cheap and this is due to the simplified joint model adopted. The increase of the computation time might not be linear in case the full model is employed.

Figure 2 shows the distribution of the Effective Sample Size across the MCMC joint posterior samples for each cell of the factorial design. Such a metric helps assessing the convergence of the MCMC sampels, but it also quantifies how much independent information there is in autocorrelated chains (Krushke, 2010). Having non-autocorrelated chains ensure to decrease the uncertainty of the estimation of posterior quantities of interest, such as credible intervals, which are useful for empirical research. As a substantiated heuristic, the higher the Effective Sample size the better.

It is computed according to:

$$\text{ESS} = \frac{N}{1 + 2\sum_{k=1}^{\infty} \text{ACF}(k)} \tag{1}$$

where N is the number of samples for a given chain, and $\text{ACF}(\cdot)$ is the autocorrelation function at lag k.

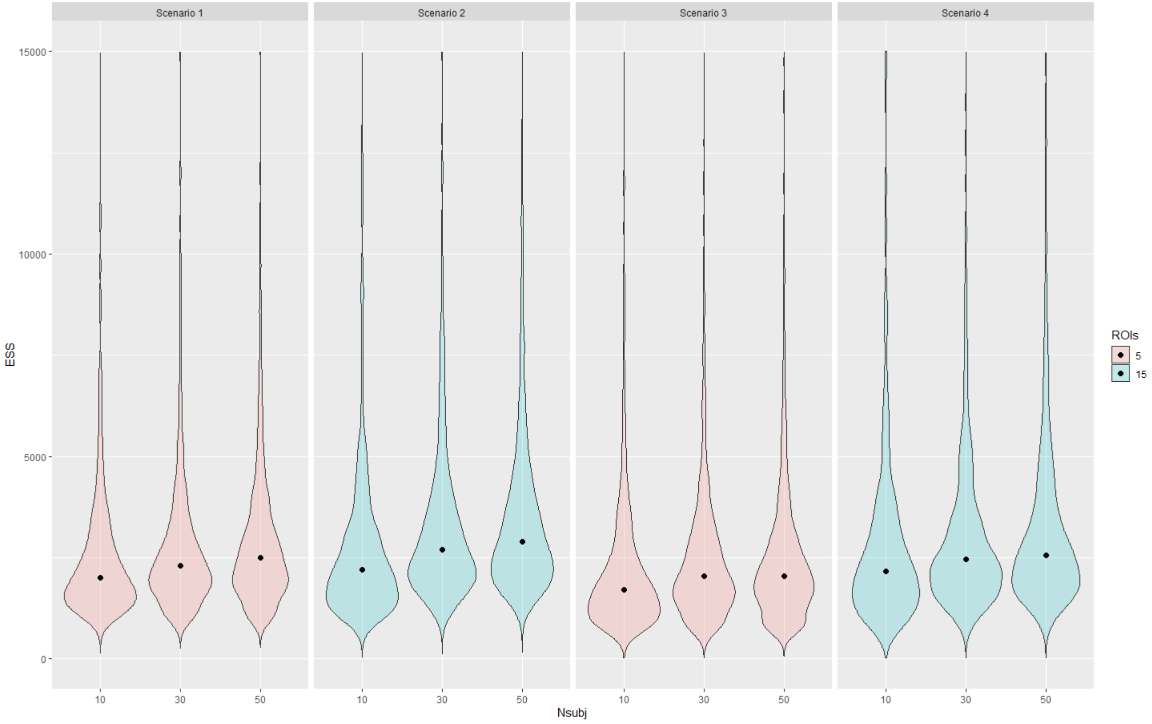

Figure 2: Effective Sample Size distribution for each cell of the factorial design. Scenarios 1 and 2 refer to fixed ζ and scenarios 3 and 4 to ζ as a free parameter.

In general, scenarios in which ζ is fixed to a default value show a higher Effective Sample Size compared to that of the scenarios in which ζ is sampled and included in the joint posterior. Enriching information yielded by the data, by increasing both sample size and ROIs, seems to ensure a more reliable posterior distributions. This provides an advantage when more data are available, due the fact that computation time seems to be affected by larger data structure to a lesser extent. In a similar way, reliability of posterior samples when ζ is assumed as a free parameter seems to be affected by the amount of data available. On the other hand, ζ seems to be generally unreliable even when larger datasets are

considered. In our case, mean Effective Sample Size estimates for ζ samples were 143(SD=18) (resp. 272(SD=34)) for 50 synthetic subjects and 5 ROIs (resp. 15 ROIs).

4 Sensitivity Analysis

In this section we provide a Sensitivity Analysis to assess the influence of several class of prior distributions on model's posterior. Due to the complexity of the hierarchical model we propose an informal approach (Roos et al., 2015) based on a factorial design in which repetitive fits of the model are performed with ad hoc modified prior inputs. We provide 7 scenarios in which several classes of prior distributions are employed for the main parameter of interest: μ_γ, μ_β, μ_{α_0}, μ_{α_1}, $\boldsymbol{\rho} = (\rho_1, \rho_2, \rho_3, \rho_4)$. The choice of relying on such a subset is justified by the fact that the high number of parameters in the model would make an exhaustive treatment of each (hyper) prior infeasible. Table 1 shows the 7 scenarios characterized by different patterns of prior specifications. In particular, in the first 4 scenario only the prior distributions of the correlation parameters in the covariance matrix of the joint model are modulated by increasing the bias towards the null hypothesis (priors increasingly peak at zero). In the remaining 3 scenarios, several patterns of priors are considered. Parameter posteriors are compared based on the 95% CI. Results are shown in Figure 1.

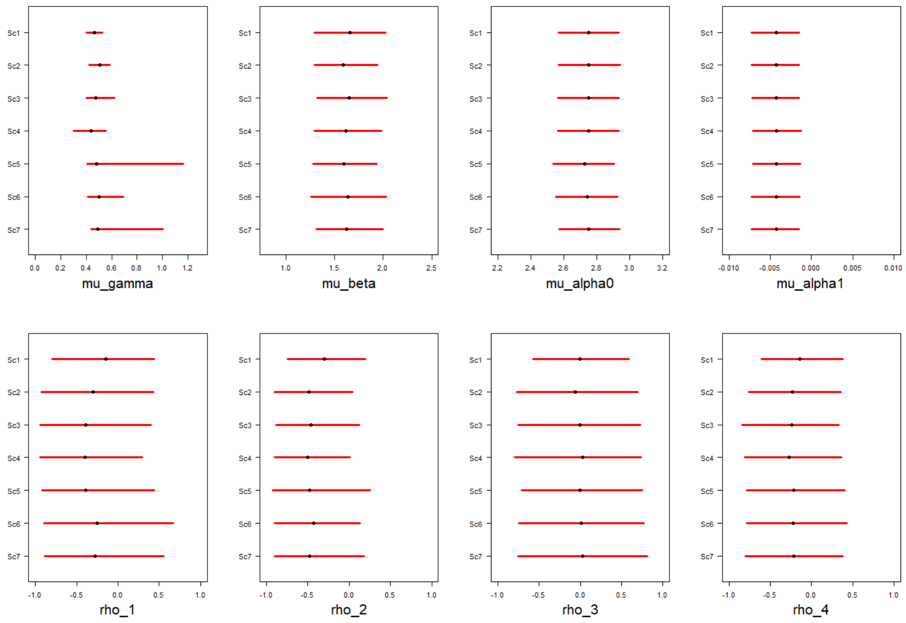

Figure 3: 95% CI (red segment) and MAP (dot) of posterior distributions of parameters of interest for each of the 7 scenarios considered. For each scenario, the joint posterior consisted of 5000 samples from 6 chains and a burn-in of 1000.

Scenario	1	2	3	4	5	6	7
μ_γ	$N(0,10^3)_{I(0,\infty)}$	$N(0,10^3)_{I(0,\infty)}$	$N(0,10^3)_{I(0,\infty)}$	$N(0,10^3)_{I(0,\infty)}$	$G(0.001,0.001)$	$U(0,5)$	$N(0,1)_{I(0,\infty)}$
μ_β	$N(0,10^3)_{I(0,\infty)}$	$N(0,10^3)_{I(0,\infty)}$	$N(0,10^3)_{I(0,\infty)}$	$N(0,10^3)_{I(0,\infty)}$	$G(0.001,0.001)$	$U(0,5)$	$N(0,1)_{I(0,\infty)}$
μ_{α_0}	$N(0,10^3)_{I(0,3)}$	$N(0,10^3)_{I(0,3)}$	$N(0,10^3)_{I(0,3)}$	$N(0,10^3)_{I(0,3)}$	$G(0.001,0.001)$	$U(0,5)$	$N(0,1)_{I(0,\infty)}$
μ_{α_1}	$N(0,1)_{I(-0.2,0.2)}$	$N(0,1)_{I(-0.2,0.2)}$	$N(0,1)_{I(-0.2,0.2)}$	$N(0,1)_{I(-0.2,0.2)}$	$N(0,1)$	$N(0,1)$	$N(0,10^3)_{I(-2,2)}$
$\boldsymbol{\rho}$	$N(0,10^3))_{I(-1,1)}$	$N(0,1))_{I(-1,1)}$	$N(0,10^{-0.2}))_{I(-1,1)}$	$N(0,10^{-1}))_{I(-1,1)}$	$U(-1,1)$	$N(0,1))_{I(-1,1)}$	$N(0,10^3))_{I(-1,1)}$

Table 1: Scenarios for Bayesian Sensitivity Analysis. N = Normal distribution, G = Gamma distribution, U = Uniform distribution.

5 JAGS Code

Here, the JAGS code of the generative model is provided:

```
model{


# BehavNeur[1] = Delta1 (ACC-Insula-IFG)

# BehavNeur[2] = Delta2 (dlPFC-Thalamus-Striatum)

# BehavNeur[3] = Gamma (risk taking)

# BehavNeur[4] = Beta (response variability)


for(s in 1:nsubj){

for(k in 1:ntrials){

omega[s,k] <- -max(0,min(5,BehavNeur[s,3]))/log(1-p[s,k])

C[s,k] ~ dbeta(a[s,k],b[s,k])

  a[s,k] <- mu_alpha[s,k] * sigma_alpha

  b[s,k] <- (1-mu_alpha[s,k])* sigma_alpha

  mu_alpha[s,k] <- 1/(1+exp(-(alpha_0[s] + alpha_1[s]*k)))

p[s,k] <- 1 - mu_alpha[s,k]

for(j in 1:Noccasion[s,k]){

theta[k,j,s] <- 1/(1 + exp(BehavNeur[s,4]*(k-omega[s,k]))

Y[k,j,s] ~ dbern(theta[k,j,s])

}

}

}


for(s in 1:nsubj){

for(r in 1:Ntracts1){

FA1[s,r] ~ dnorm(BehavNeur[s,1],1/pow(sigma_1,2))

}

for(u in 1:Ntracts2){
```

```
FA2[s,u] ~ dnorm(BehavNeur[s,2],1/pow(sigma_2,2))
}
}

for(s in 1:nsubj){
BehavNeur0[s,1:4] ~ dmnorm.vcov(zeros[], cov[1:4,1:4])
scale[s] ~ dchisq(df)
BehavNeur[s,1] <- mu[1] + BehavNeur0[s,1]*sqrt(df/scale[s])
BehavNeur[s,2] <- mu[2] + BehavNeur0[s,2]*sqrt(df/scale[s])
BehavNeur[s,3] <- mu[3] + BehavNeur0[s,3]*sqrt(df/scale[s])
BehavNeur[s,4] <- mu[4] + BehavNeur0[s,4]*sqrt(df/scale[s])
alpha_0[s] ~ dnorm(mu_alpha_0, 1/pow(sigma_alpha_0,2))
alpha_1[s] ~ dnorm(mu_alpha_1,1/pow(sigma_alpha_1,2))
}

zeros[1] <- 0
zeros[2] <- 0
zeros[3] <- 0
zeros[4] <- 0

cov[1,1] <- sigma[1]*sigma[1]
cov[1,2] <- 0
cov[1,3] <- sigma[1]*sigma[3]*rho1
cov[1,4] <- sigma[1]*sigma[4]*rho2

cov[2,1] <- 0
cov[2,2] <- sigma[2]*sigma[2]
cov[2,3] <- sigma[2]*sigma[3]*rho3
cov[2,4] <- sigma[2]*sigma[4]*rho4
```

```
cov[3,1] <- sigma[1]*sigma[3]*rho1
cov[3,2] <- sigma[2]*sigma[3]*rho3
cov[3,3] <- sigma[3]*sigma[3]
cov[3,4] <- 0

cov[4,1] <- sigma[1]*sigma[4]*rho2
cov[4,2] <- sigma[2]*sigma[4]*rho4
cov[4,3] <- 0
cov[4,4] <- sigma[4]*sigma[4]

# Priors

mu[1] ~ dnorm(0,0.001)
mu[2] ~ dnorm(0,0.001)
mu[3] ~ dnorm(0,0.001)T(0,)
mu[4] ~ dnorm(0,0.001)T(0,)
sigma[1] ~ dgamma(0.01,0.01)
sigma[2] ~ dgamma(0.01,0.01)
sigma[3] ~ dgamma(0.01,0.01)
sigma[4] ~ dgamma(0.01,0.01)
rho1 ~ dunif(-0.99,0.99)
rho2 ~ dunif(-0.99,0.99)
rho3 ~ dunif(-0.99,0.99)
rho4 ~ dunif(-0.99,0.99)
sigma_1 ~ dgamma(0.01,0.01)
sigma_2 ~ dgamma(0.01,0.01)
mu_alpha_0 ~ dnorm(0,0.001)T(0,3)
sigma_alpha_0 ~ dunif(0,3)
```

```
mu_alpha_1 ~ dnorm(0,1)T(-0.2,0.2)

sigma_alpha_1 ~ dunif(0,1)

sigma_alpha ~ dunif(0,100)

df <- 5


}
```

6 References

Kruschke, J. K. (2010). Doing Bayesian data analysis: A tutorial with R and BUGS. New York, NY: Academic Press.

Roos, M., Martins, T. G., Held, L., and Rue, H. (2015a). Sensitivity analysis for Bayesian hierarchical models. *Bayesian Analysis*, 10(2):321–349.
